# Daratumumab combined with anti-CD20 therapy in pediatric and adult refractory idiopathic nephrotic syndrome: single-center experience

**DOI:** 10.3389/fimmu.2025.1722023

**Published:** 2025-12-02

**Authors:** Hamza Naciri Bennani, Thomas Sandaye, Quentin Charroyer, Aurelie Etangsale, Henri Vacher Coponat, Marie Julien, Vincent Mear, Olivier Dunand, Ludovic Di Ascia

**Affiliations:** 1 Nephrology, Hemodialysis, Apheresis and Kidney Transplantation Department, Felix Guyon University Hospital, Saint Denis La Reunion, France; 2 Clinical Research and Innovation Unit, Felix Guyon University Hospital, Saint Denis La Reunion, France; 3 University of Reunion, Faculty of Medicine, Saint Denis La Reunion, France; 4 Pharmacy Department, Felix Guyon University Hospital, Saint Denis La Reunion, France; 5 Pediatric Nephrology Department, Felix Guyon University Hospital, Saint Denis La Reunion, France

**Keywords:** idiopathic nephrotic syndrome, daratumumab, plasma cells, anti-CD20, refractory nephrotic syndrome, kidney transplantation

## Abstract

**Background:**

Refractory idiopathic nephrotic syndrome (INS), in native kidneys or post-transplant, represents a major therapeutic challenge due to its high risk of progressing to end-stage renal disease. Patients often resist anti-CD20 therapy and require prolonged apheresis. Daratumumab, an anti-CD38 monoclonal antibody targeting plasma cells, has demonstrated promising efficacy in case reports when combined with anti-CD20, but evidence remains limited.

**Methods:**

We conducted a single-center retrospective study including four patients (two pediatric, two adult, including one renal transplant) with INS refractory to conventional therapies (corticosteroids, calcineurin inhibitors, mycophenolate mofetil, anti-CD20 antibodies, and/or apheresis). All Patients received daratumumab combined with anti-CD20 therapy at University Hospital of La Réunion between 2022 and 2025. Clinical and laboratory data were extracted from medical records. Renal response was defined as complete remission (CR) or partial remission (PR).

**Results:**

The mean patient age was 20 ± 13 years (range: 8–40), with a male-to-female ratio of 3:1. Median follow-up after daratumumab administration was 5 months (range: 2–12). All patients achieved CR with a median time to response of 25 days (range: 14–30). Previously apheresis-dependent patients were able to discontinue sessions. Two patients developed infections (herpetic and SARS-CoV-2 pneumonia complicated by pneumococcal bacteremia), all resolving favorably. Renal function remained stable.

**Conclusion:**

Combined daratumumab and anti-CD20 therapy appears to be an effective rescue strategy for refractory INS, in native kidneys and post-transplant. It induces rapid and sustained remission, enabling discontinuation of apheresis. Prospective studies are warranted to optimize treatment regimens and identify predictive biomarkers of response.

## 1 Introduction

Idiopathic nephrotic syndrome (INS) is an acquired glomerular disorder characterized by disruption of the glomerular filtration barrier, leading to massive proteinuria. It is the leading cause of glomerular disease in children and also affects adults, encompassing mainly minimal change disease (MCD) and focal segmental glomerulosclerosis (FSGS) (1–3). While MCD usually presents as a steroid-sensitive form with favorable outcomes, FSGS is more often steroid-resistant and associated with progressive renal impairment (4–8).

The pathophysiology of INS involves podocyte injury secondary to dysregulated immune mechanisms, including circulating permeability factors such as soluble urokinase plasminogen activator receptor (suPAR), cardiotrophin-like cytokine-1, soluble CD40 ligand, IL-13, hemopexin, angiopoietin-like 4, and, more recently, anti-nephrin antibodies detected in nearly half of patients (9–16). Altogether, these data support the concept that immune dysregulation involving B cells, plasma cells, and antibody-mediated mechanisms plays a central role in disease persistence and recurrence.

Despite corticosteroids and second-line agents such as calcineurin inhibitors, mycophenolate mofetil, or anti-CD20 antibodies, 10–20% of patients remain steroid- or multi-drug-resistant, exposing them to a high risk of progression to end-stage kidney disease in nearly 40–50% of cases within 6 to 8 years (17–23). A meta-analysis including 423 patients from 77 studies reported that plasmapheresis achieved remission in approximately 70% of children and 63% of adults, although a significant proportion of patients remain refractory or relapse after treatment discontinuation (24, 25). In kidney transplantation, primary FSGS frequently recurs early after grafting, sometimes immediately post-reperfusion, with recurrence rates of approximately 30% after the first transplant and approaching 100% in subsequent transplants, despite intensified immunosuppression (26–30). Despite this significant risk, transplantation remains indicated in these patients, particularly due to their young age.

In this context, there is an urgent need for novel therapeutic approaches. Plasma cell-targeting strategies, such as daratumumab—a monoclonal anti-CD38 antibody initially developed for multiple myeloma—have recently shown promising efficacy in several reported cases of refractory INS, both in native kidneys and post-transplant, in combination with anti-CD20 antibodies (31, 32). This combined approach allows for synergistic depletion of B lymphocytes and plasma cells, offering a more durable immunological remission.

Here, we report the experience of the University Hospital of La Réunion in treating four patients—two children and two adults—with refractory INS managed with daratumumab-based regimen combined with anti-CD20 therapy, either in native kidneys or following transplantation.

## 2 Patients and methods

### 2.1 Study population

We conducted a single-center retrospective study including four patients (two adults and two children) with refractory idiopathic nephrotic syndrome (INS) treated with daratumumab at the University Hospital of La Réunion between January 2022 and August 2025.

The diagnosis of INS was confirmed by renal biopsy in two patients (patients 2 and 4), revealing minimal change disease (MCD). The remaining two patients (patients 1 and 3) did not undergo biopsy. Genetic testing was performed for all patients and yielded negative results.

All patients had previously received conventional therapy, including corticosteroids, calcineurin inhibitors, mycophenolate mofetil, anti-CD20 antibodies (rituximab and/or obinutuzumab), and/or apheresis, yet remained either therapy-dependent, frequently relapsing, or non-responsive.

The following definitions were applied:

- Relapse: urinary protein-to-creatinine ratio (UPCR) > 300 mg/mmol (in adults) or > 200 mg/mmol (in pediatrics) after complete or partial remission.
- Frequent relapses: ≥ 2 relapses within 6 months or ≥ 4 relapses within 12 months following initial remission.
- Steroid-dependence: relapse during tapering of corticosteroids or within 2 weeks of cessation.
- Steroid-resistance: failure to achieve remission after 16 weeks of corticosteroid therapy at 1 mg/kg/day in adults, and after 4 weeks of oral prednisone at 60 mg/m²/day followed by 10 days after three intravenous methylprednisolone pulses (1 g/1.73 m²) in pediatric patients.
- Multi-drug resistant: absence of complete remission after 12 months of treatment with two mechanistically distinct steroid-sparing agents at standard doses (33).
- Apheresis-resistant: absence of complete remission after one month of standard apheresis therapy.
- Apheresis-dependent: relapse occurs upon cessation of apheresis or when the interval between sessions is extended.
- Post-transplant FSGS recurrence: UPCR > 300 mg/mmol after exclusion of other post-transplant causes, particularly acute antibody-mediated rejection (evaluated by donor-specific antibodies, serum creatinine trends, and urine output).

### 2.2 Endpoints

The primary endpoint was renal response to daratumumab:

- Complete remission (CR): UPCR < 30 mg/mmol and serum albumin > 30 g/L.
- Partial remission (PR): serum albumin > 30 g/L with UPCR 30–350 mg/mmol, or a ≥50% reduction from baseline.

Secondary endpoints included changes in renal function, serum albumin, treatment tolerance, and occurrence of opportunistic infections.

### 2.3 Treatment protocol

Daratumumab was administered either intravenously (16 mg/kg) or subcutaneously (1800 mg) in combination with anti-CD20 therapy and apheresis, except for Patient 1 who could not receive apheresis due to living on Mayotte. In the absence of established guidelines, dosing frequency and schedule were determined by the treating physician based on clinical and laboratory response.

For the transplant recipient, maintenance immunosuppression included tacrolimus, mycophenolate mofetil, and prednisone following induction with antithymocyte globulin. Prophylactic antibiotics included trimethoprim-sulfamethoxazole, phenoxymethylpenicillin, and valacyclovir. All patients were up to date on vaccinations, including pneumococcal immunization.

### 2.4 Collected data and statistical analyses

Clinical and laboratory data were extracted from electronic medical records and hospital reports. Follow-up parameters included serum creatinine, serum albumin, UPCR, and lymphocyte counts. Adverse events related to daratumumab infusions were recorded.

Continuous variables are reported as mean ± standard deviation or median (range), and categorical variables as counts and percentages. Statistical analyses were performed using GraphPad Prism^®^ v10.

All patients were informed and did not oppose the use of biological samples and clinical data for research purposes. Informed consent for publication of this case series was obtained. As this is a retrospective case series, formal ethics committee approval was not required according to institutional policy.

## 3 Results

Four patients with idiopathic nephrotic syndrome—two children (8 and 15 years old) and two adults (19 and 40 years old) —were treated with daratumumab in combination with anti-CD20 therapies (obinutuzumab, rituximab) and apheresis techniques (immunoadsorption, double filtration plasmapheresis [DFPP]). The mean age was 20 ± 13 years (range 8–40), with a male-to-female ratio of 3:1. All patients had steroid-dependent or multi-drug refractory disease, having previously received multiple lines of therapy (calcineurin inhibitors, mycophenolate mofetil). Three patients had native kidney disease, while one adult female experienced early post-transplant recurrence on day 3.

**Figure 1:**
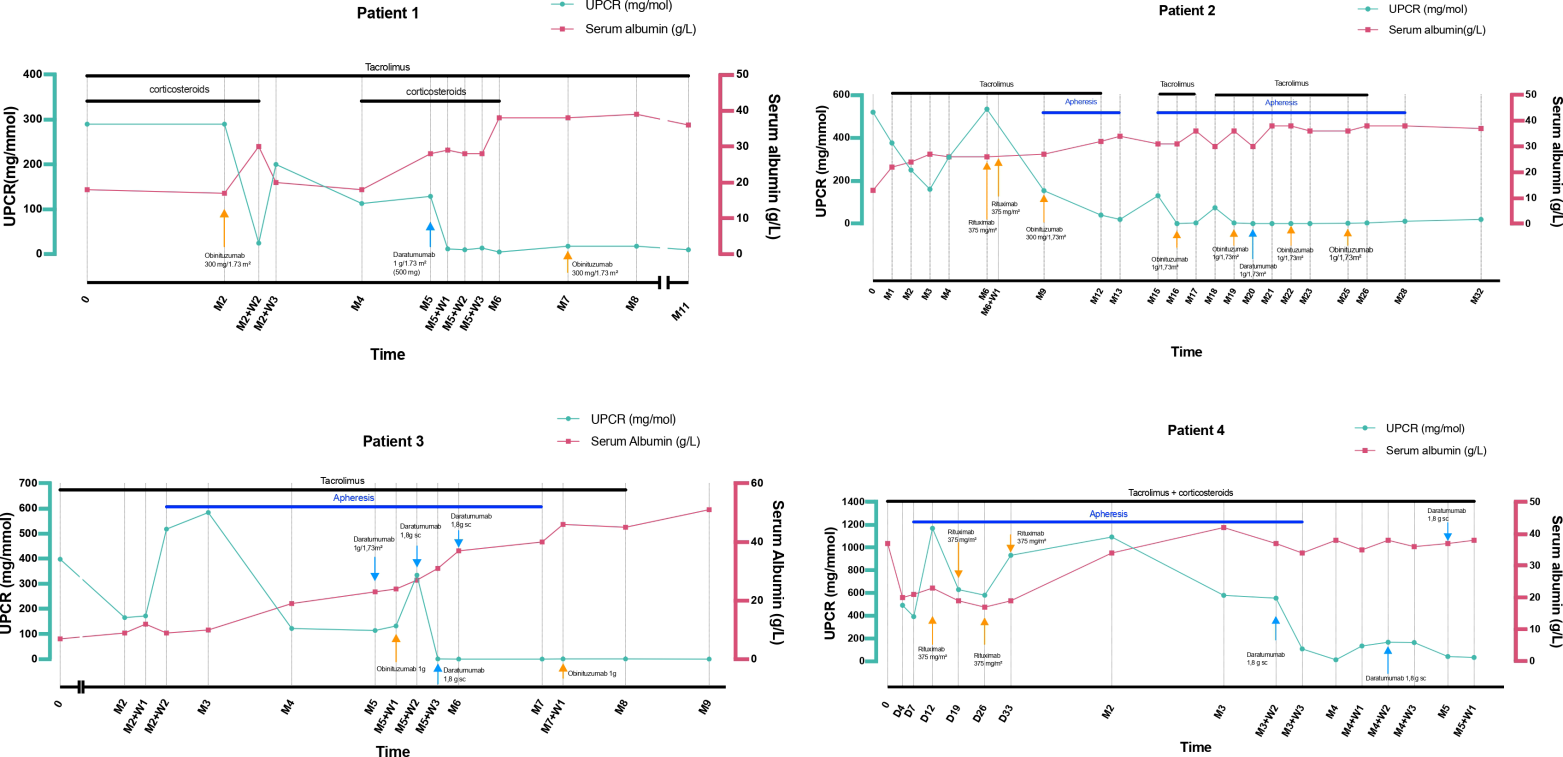
Serum albumin (g/L) and urinary protein-to-creatinine ratio (mg/mmol) outcomes during follow-up. D, day; M, Month; SC, Subcutaneous; UPCR, Urinary protein-to-creatinine ratio; W, Week.

**Figure 2:**
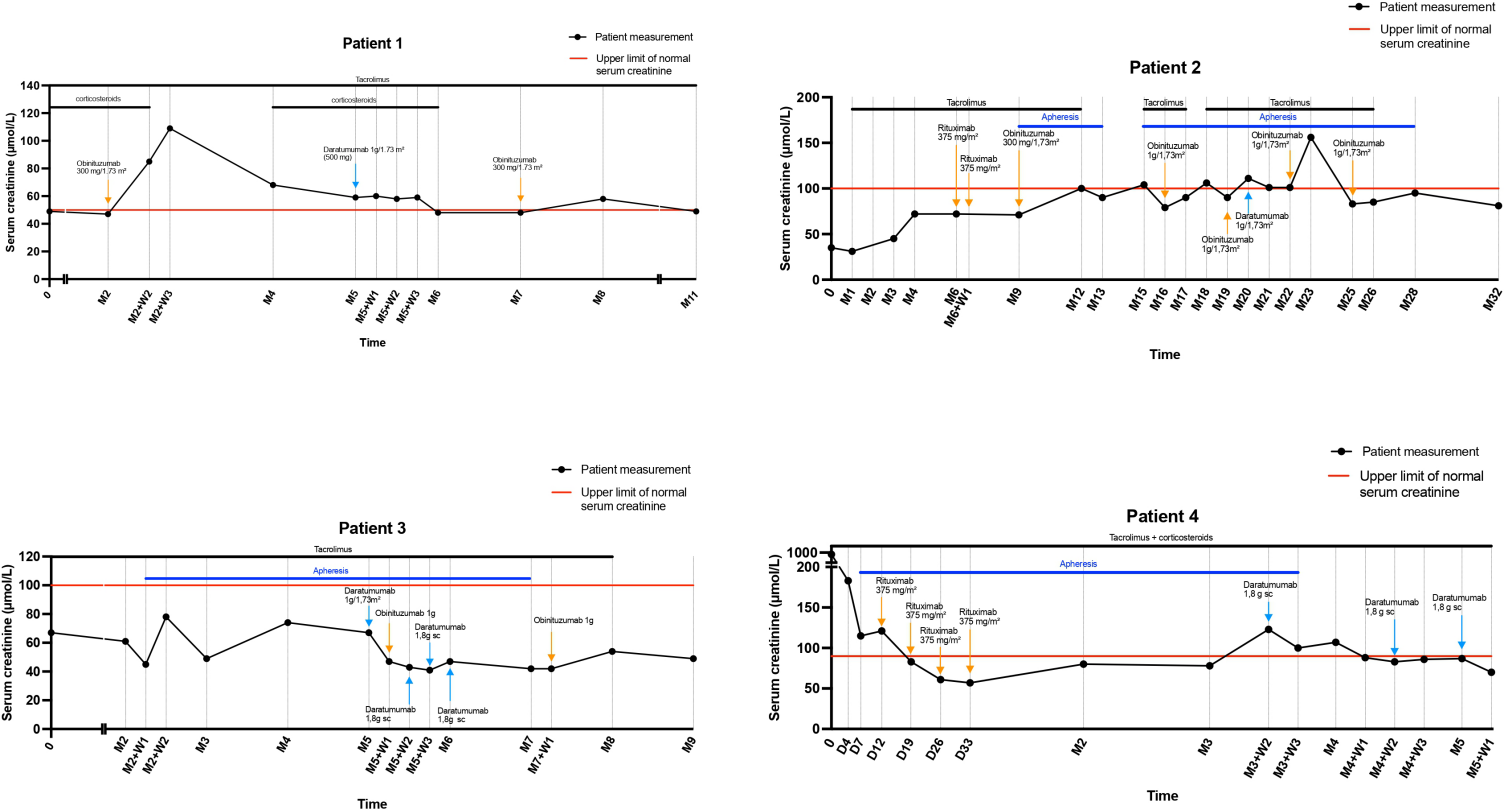
Serum creatinine (μmol/L) outcomes during follow-up. D, day; M, Month; SC, Subcutaneous; W, Week.

**Figure 3:**
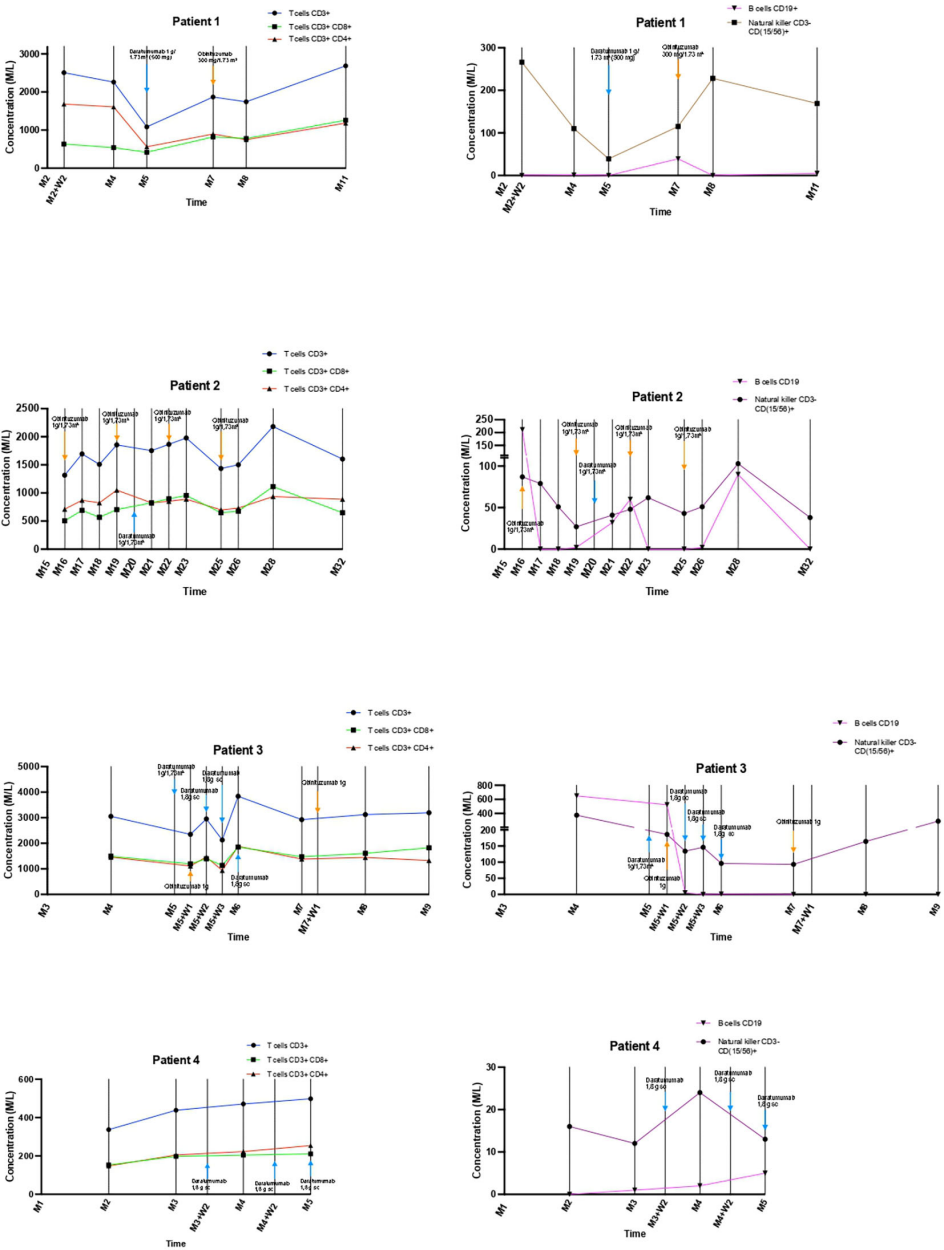
Lymphocyte and NK cell dynamics during follow-up.

Combined daratumumab and anti-CD20 therapy induced complete remission (CR) in all four patients, achieved after a median of 25 days (range 14–30) following the first daratumumab infusion (**Figure 1**), sometimes consolidated with apheresis. Median follow-up after the first daratumumab infusion was 5 months (range 2–12). Renal function, as assessed by serum creatinine, remained stable throughout the follow-up period (**Figure 2**). Immune monitoring data for lymphocyte and NK-cell subsets are presented in **Figure 3**.

Two patients experienced infectious complications, including herpetic infections and SARS-CoV-2 pneumonia complicated by pneumococcal bacteremia, all resolving favorably, whereas the remaining two patients had no notable infectious events. Clinical characteristics and outcomes of patients treated with daratumumab combined with anti-CD20 therapy for refractory idiopathic nephrotic syndrome are summarized in **Table 1**.

**Table 1:** Baseline characteristics and outcomes of patients treated with daratumumab and anti-CD20 therapy for refractory INS.

Patient	Age (years)/Sex (M/F)	Native kidney/Transplant	Number of previous relapses	Previous therapies	Clinical status before daratumumab	Last-line therapy before daratumumab	Daratumumab regimen (IV/SC)	Baseline renal parameters before Daratumumab	Renal response	Time to CR*	Outcome at last follow-up	Complications
1	8/M	Native kidney	7	Corticosteroids, tacrolimus, MMF, rituximab, obinutuzumab	steroid-dependent, multi-drug-resistant	Obinutuzumab (M-3), tacrolimus, corticosteroids	IV 500 mg	Alb 28 g/L, UPCR 129 mg/mmol, Cr 59 µmol/L	CR	1 month	CR maintained at 6 months (Alb 36 g/L, UPCR 10 mg/mmol, Cr 49 µmol/L)	Herpes infections, stomatitis, febrile aplasia, transient toxic anemia
2	15/M	Native kidney	5	Corticosteroids, Tacrolimus, rituximab, obinutuzumab, DFPP, IA	Steroid-resistant, Apheresis-dependent	Obinutuzumab (M-1), IA, tacrolimus	IV 1000 mg/1.73 m²	Alb 30 g/L, UPCR 0,71 mg/mmol, Cr 111 µmol/L	CR	1 month	Sustained CR at 12 months (Alb 37 g/L, UPCR 20,03 mg/mmol, Cr 101 µmol/L), IA withdrawal achieved	None
3	19/M	Native kidney	8	Corticosteroids, MMF, tacrolimus, rituximab, obinutuzumab, IA, DFPP	Steroid-dependent, multi-drug-resistant, Apheresis-resistant	Obinutuzumab (W + 1), IA, tacrolimus	IV 1000 mg ×1 then SC 1800 mg ×3	Alb 23 g/L, UPCR 115 mg/mmol, Cr 67 µmol/L	CR	3 weeks	CR maintained at 4 months (Alb 51 g/L, UPCR 1.18 mg/mmol, Cr 49 µmol/L), IA withdrawal achieved	COVID-19 pneumonia with pneumococcal bacteremia
4	40/F	Transplant	1	Corticosteroids, tacrolimus, MMF, rituximab, IA	Early post-transplant recurrence (day 3), multi-drug-resistant, Apheresis-dependent	Rituximab (M-2), IA, tacrolimus, MMF, corticosteroids	SC 1800 mg ×3	Alb 37 g/L, UPCR 555 mg/mmol, Cr 123 µmol/L	PR at 1 week, CR at 2 weeks	2 weeks	CR at 2 months (Alb 37 g/L, UPCR 25 mg/mmol, Cr 70 µmol/L), IA withdrawal achieved	None

INS, Idiopathic Nephrotic Syndrome; CR, complete remission; PR, partial remission; Alb, serum albumin; UPCR, urinary protein-to-creatinine ratio; Cr, serum creatinine; MMF, mycophenolate mofetil; IA, immunoadsorption; DFPP, double filtration plasmapheresis; M, Male; F, Female; M, Months; W, Week.

### 3.1 Patient 1: steroid-dependent, multi-drug-resistant

An 8-year-old boy with steroid-dependent INS diagnosed in 2020 experienced multiple relapses despite treatment with corticosteroids, tacrolimus, mycophenolate mofetil, and rituximab.

In September 2024, he presented with a relapse complicated by acute kidney injury and hypertension, requiring intensified tacrolimus and corticosteroid therapy. Two months later, another relapse occurred ten days after corticosteroid discontinuation and three weeks after an initial obinutuzumab infusion (300 mg/1.73 m²), which achieved B-cell depletion by day 15. Corticosteroid taper was intentionally accelerated for steroid-sparing purposes, given the prolonged exposure from previous relapses.

Consequently, a first daratumumab infusion (500 mg IV) was administered in February 2025 and was well tolerated. At that time, laboratory parameters included serum albumin of 28 g/L, UPCR of 129 mg/mmol, and serum creatinine of 59 µmol/L. Complete remission was achieved one month later, with serum albumin of 38 g/L, UPCR of 5 mg/mmol, and serum creatinine of 48 µmol/L, allowing discontinuation of corticosteroids.

A second obinutuzumab infusion (300 mg/1.73 m²) was administered two months after daratumumab to consolidate the response. At six months, the patient remained in CR, with serum albumin of 36 g/L, UPCR of 10 mg/mmol, and serum creatinine of 49 µmol/L.

Infectious complications included auricular herpes, herpetic stomatitis, and febrile neutropenia of unknown origin, all resolving with antibiotics and G-CSF therapy. The patient also developed likely drug-induced anemia, requiring temporary treatment with erythropoietin beta.

### 3.2 Patient 2: steroid-resistant, apheresis-dependent

A 15-year-old adolescent was diagnosed in 2021 with steroid-resistant INS, presenting with serum albumin of 13 g/L, UPCR of 519 mg/mmol, and serum creatinine of 35 µmol/L. Tacrolimus induced a partial remission at month 2, which was rapidly complicated by renal toxicity.

Six months after diagnosis, two rituximab infusions (375 mg/m²) administered one week apart failed to induce complete remission (serum albumin 27 g/L, UPCR 154 mg/mmol). Three months later, treatment with DFPP (33 sessions over five months), combined with obinutuzumab (300 mg/1.73 m²) and tacrolimus, achieved CR (serum albumin 34 g/L, UPCR 20 mg/mmol). However, four months after DFPP discontinuation, UPCR increased to 130 mg/mmol, requiring resumption of apheresis (immunoadsorption) and tacrolimus, followed by two additional obinutuzumab infusions (1,000 mg/1.73 m² each, spaced three months apart).

Following national multidisciplinary team discussion and despite the prior relapse after apheresis withdrawal under obinutuzumab therapy, intravenous daratumumab (1,000 mg/1.73 m²) was administered in April 2023 with the aim of discontinuing apheresis, despite serum albumin was 30 g/L, UPCR 0.71 mg/mmol, and serum creatinine 111 µmol/L. One month after daratumumab administration, serum albumin normalized to 38 g/L, UPCR decreased to 0.34 mg/mmol, and serum creatinine to 101 µmol/L. Immunoadsorption was discontinued in January 2024 after 45 sessions, with stable CR maintained.

At 12-months follow-up post-daratumumab combined with obinutuzumab, the response remained durable (serum albumin 37 g/L, UPCR 20 mg/mmol, serum creatinine 81 µmol/L). Two additional obinutuzumab infusions (1000 mg/1.73 m²) were administered after daratumumab to consolidate the response. No infectious complications were observed.

### 3.3 Patient 3: steroid-dependent, multi-drug-resistant, apheresis-resistant

A 19-year-old man with steroid-sensitive, pharmacodependent INS diagnosed in 2010 experienced multiple relapses treated with corticosteroids, mycophenolate mofetil, ciclosporin, tacrolimus, and rituximab. In August 2021, a severe relapse required a combined protocol of apheresis, daratumumab (1000 mg/1.73 m² IV weekly ×4), and obinutuzumab (1000 mg at month 0, then at months 3 and 12), together with tacrolimus, resulting in CR in March 2022, maintained by obinutuzumab maintenance (1000 mg) in March 2023.

In July 2024, the patient experienced an eighth relapse (serum albumin 7 g/L, UPCR 398 mg/mmol, serum creatinine 67 µmol/L). Tacrolimus was reintroduced, but the patient was lost to follow-up until December 2024, when he presented with severe nephrotic syndrome (serum albumin 9 g/L, UPCR 166 mg/mmol, serum creatinine 61 µmol/L). Immunoadsorption (IA) was initiated in January 2025 in combination with high-dose calcineurin inhibitors, but no remission was achieved after 50 sessions performed over approximately two months (serum albumin 19 g/L, UPCR 123 mg/mmol, serum creatinine 74 µmol/L).

In March 2025, a daratumumab induction regimen was started (1000 mg/1.73 m²) IV followed by three subcutaneous injections of 1800 mg on days 14, 21, and 28 due to IV infusion intolerance with tachycardia, headache, hypotension, and vomiting) in combination with an obinutuzumab infusion (1000 mg) on day 7.

Complete remission was achieved just before the third daratumumab injection (serum albumin 31 g/L, UPCR 1.5 mg/mmol, serum creatinine 41 µmol/L). After daratumumab initiation, IA was maintained for an additional 17 sessions over two months—three sessions per week during the first month, then one per week during the second—to prevent early relapse and consolidate remission, and was finally discontinued in May 2025 once stable complete remission was confirmed (serum albumin 40 g/L, UPCR 1.28 mg/mmol, serum creatinine 42 µmol/L). A second obinutuzumab infusion (1000 mg) was administered one week after IA discontinuation, with calcineurin inhibitor tapering two months later.

At four months post-daratumumab, CR was confirmed off therapy (serum albumin 51 g/L, UPCR 1.18 mg/mmol, serum creatinine 49 µmol/L). The course was complicated by SARS-CoV-2 pneumonia with pneumococcal bacteremia, requiring one-week hospitalization and treatment with ceftriaxone and Paxlovid, with a favorable outcome and no sequelae.

### 3.4 Patient 4: multi-drug-resistant, apheresis-dependent

A 40-year-old woman followed since 1988 for steroid-resistant INS experienced multiple relapses during her therapeutic course, treated sequentially with corticosteroids, chlorambucil, ciclosporin, cyclophosphamide, mycophenolate mofetil, and tacrolimus, achieving only partial and transient remissions. The disease course eventually led to end-stage renal disease requiring dialysis in 2021.

She underwent a first kidney transplant in March 2025 from a deceased donor, with immediate graft function recovery. Induction therapy included thymoglobulin, corticosteroids, tacrolimus, and mycophenolate mofetil. An early INS recurrence was observed on day 3 post-transplant (serum albumin 20 g/L, UPCR 493 mg/mmol).

An intensive immunoadsorption (IA) protocol (five sessions per week for four weeks, then spaced out), combined with four rituximab infusions (375 mg/m² at one-week intervals), did not achieve complete remission. A subcutaneous daratumumab infusion (1800 mg) was administered 45 days after the last rituximab infusion as part of a combined anti-CD20/anti-CD38 approach.

Three weeks later, proteinuria reappeared (UPCR 134 mg/mmol) and subsequently increased to 168 mg/mmol, justifying two additional subcutaneous daratumumab injections (1800 mg each) spaced two weeks apart. One week after the third injection, the patient achieved CR (serum albumin 38 g/L, UPCR 34 mg/mmol, serum creatinine 70 µmol/L).

At the last follow-up, two weeks after the third daratumumab infusion, renal function remained stable (serum creatinine 70 µmol/L, UPCR 25.39 mg/mmol, serum albumin 37 g/L). No infectious complications were reported.

## 4 Discussion

Our single-center series illustrates the therapeutic potential of the daratumumab and anti-CD20 combination in refractory forms of idiopathic nephrotic syndrome (INS), both in native kidneys and in post-transplant recurrence. In our four patients — two children (8 and 15 years old) and two adults (19 and 40 years old) — this strategy induced complete remission after a median of 25 (7–30) days following the first daratumumab infusion, sometimes consolidated by a few apheresis sessions.

Patient 1 achieved complete remission despite the absence of apheresis, underscoring the potential efficacy of the daratumumab–anti-CD20 combination alone. Corticosteroid tapering was intentionally accelerated to minimize prolonged corticosteroid exposure, which may have contributed, at least partially, to the relapse that occurred three weeks after the initial obinutuzumab infusion. Patient 2, initially dependent on apheresis, was able to discontinue it while maintaining complete remission four months after cessation. Patient 3, an adult with multidrug-resistant and apheresis-refractory disease, achieved complete remission one month after initiating daratumumab, with additional immunoadsorption sessions mainly intended to consolidate the response. Finally, Patient 4 demonstrated that early post-transplant recurrence can also respond rapidly to the combined therapy, achieving complete remission after three daratumumab infusions and allowing discontinuation of immunoadsorption.

These observations are consistent with published data. Angeletti et al. (31) described five post-transplant patients refractory to rituximab and plasmapheresis who achieved complete remission with rituximab–daratumumab combination therapy, allowing plasmapheresis discontinuation. Disease activity correlated with circulating CD38+ plasma cells, and daratumumab retained efficacy as monotherapy during relapses occurring approximately three months after the initial combination response (31). Randone et al. (32) reported two post-transplant INS patients refractory to anti-IL1 therapy, rituximab, and plasmapheresis, treated with obinutuzumab–daratumumab, achieving complete remission in one and partial remission in the other, with apheresis discontinuation. A relapse associated with increased CD38+ plasma cells responded to an additional daratumumab dose, suggesting that resistance may result from incomplete depletion of memory B cells or plasma cells (32). Similarly, Delbet et al. (34) described a pediatric case of immediate post-transplant recurrence resistant to immunoadsorption, achieving durable complete remission after obinutuzumab–daratumumab therapy, despite *Pneumocystis jirovecii* pneumonia and persistent hypogammaglobulinemia. In 14 children with resistant INS, Dossier et al. (35) reported a 60% relapse-free survival rate at 24 months following obinutuzumab–daratumumab combination therapy, with an acceptable safety profile and only mild transient neutropenia in two patients. Immunoglobulin replacement and monitoring were required in most cases, without severe infections. No patient relapsed during the B-cell depletion phase (35).

Taken together, these data and our observations support the concept that targeting CD38+ plasma cells in combination with B-cell depletion represents an effective rescue strategy, capable of inducing rapid and sustained remission in multidrug-resistant or relapsing INS, reducing the need for prolonged apheresis, improving quality of life, and preserving renal function (24–27, 31, 32, 34–36).

### 4.1 Heterogeneity and confounding factors

Our cohort displayed marked heterogeneity: children (8 and 15 years) and adults (19 and 40 years), INS on native kidneys or post-transplant, diverse prior therapies, and use of concomitant interventions such as apheresis, which was employed in three patients initially to induce remission and subsequently to consolidate it. These differences limit generalizability and make it difficult to attribute remission exclusively to the daratumumab–anti-CD20 combination. However, the rapid achievement of complete remission in all patients, including the one who did not undergo apheresis, suggests a significant therapeutic effect of the daratumumab–anti-CD20 association.

### 4.2 Mechanistic considerations and data-driven implications

The efficacy of daratumumab can be explained by its targeting of long-lived CD38+ plasma cells, which are resistant to anti-CD20 therapy and produce antibodies, including anti-nephrin antibodies implicated in podocyte injury (9–16, 37, 38). Combination with anti-CD20 therapy depletes naïve and memory B cells as well as short-lived plasmablasts, preventing early B-cell reconstitution and promoting deeper, more sustained remission (31, 32, 34, 35, 39). Since CD38 is also expressed on activated T cells, daratumumab may exert immunomodulatory effects on T-cell subsets (39, 40). Notably, sustained depletion of CD38^+^CD19^+^ B cells has been associated with clinical response to daratumumab (41).

In refractory nephrotic syndrome, early expansion of class-switched memory B cells is consistently linked to relapse, underscoring their central role in maintaining immune activity (42). In patients receiving anti-CD20 therapy, higher baseline memory B-cell counts are associated with increased relapse risk and faster recovery of these cells after treatment, indicating that baseline memory B-cell levels are key predictors of immune reconstitution and treatment response (43). Obinutuzumab, through enhanced antibody-dependent cytotoxicity and caspase-independent direct apoptosis, induces deeper and more sustained B-cell depletion than rituximab and has shown particular benefit in rituximab-resistant podocytopathies (39, 44).

These clinical correlations, observed in both transplanted and native kidney patients, suggest that this dual-targeted approach may reduce the duration and intensity of apheresis and favor sustained remission.

### 4.3 Tolerance and safety

Tolerance to the combination in our cohort was acceptable. Two patients experienced infectious complications (herpes, SARS-CoV-2 pneumonia with bacteremia), all of which resolved favorably, and one patient developed transient anemia requiring temporary treatment with erythropoietin beta. No permanent discontinuation of therapy was required. These observations are consistent with published data, in which hypogammaglobulinemia and infection risk represent the main adverse events (34, 35, 39), requiring adapted monitoring and prophylaxis.

### 4.4 Limitations and perspectives

The main limitations of our study include the small sample size, relatively short follow-up (median 5 months), and heterogeneity of patients and interventions, which complicate assessment of long-term remission durability and safety. Co-interventions (apheresis) also represent confounding factors. Nevertheless, our results suggest a promising role for the daratumumab–anti-CD20 combination as a salvage therapy in multidrug-resistant and relapsing INS. Prospective multicenter studies are warranted to define optimal dosing, administration route, and consolidation/maintenance strategies, and to identify predictive biomarkers of response (circulating CD38+ plasma cells, anti-nephrin antibodies) to guide patient management and follow-up.

## 5 Conclusion

Our case series highlights that the combination of daratumumab and anti-CD20 therapy represents an innovative and effective therapeutic option for refractory INS, capable of inducing rapid and sustained complete remission, enabling discontinuation of apheresis, and improving quality of life, both in native kidneys and post-transplant. Despite the limited sample size and clinical heterogeneity, these consistent therapeutic responses support the integration of this approach into salvage treatment strategies and underscore the need for prospective multicenter studies to refine treatment protocols and identify predictive biomarkers of response.

## Data Availability

The original contributions presented in the study are included in the article/supplementary material. Further inquiries can be directed to the corresponding author.
